# The prevalence of childhood trauma in psychiatric outpatients

**DOI:** 10.1186/s12991-019-0239-1

**Published:** 2019-08-14

**Authors:** Fiona Devi, Shazana Shahwan, Wen Lin Teh, Rajeswari Sambasivam, Yun Jue Zhang, Ying Wen Lau, Say How Ong, Daniel Fung, Bhanu Gupta, Siow Ann Chong, Mythily Subramaniam

**Affiliations:** 1 Research Division, Institute of Mental Health, Buangkok Green Medical Park, 10 Buangkok View, Singapore, 539747 Singapore; 2 Department of Mood & Anxiety, Institute of Mental Health, Singapore, Singapore; 3 Department of Developmental Psychiatry, Institute of Mental Health, Singapore, Singapore

**Keywords:** Outpatients, Prevalence, CTQ, Psychotic disorder, Mood disorder

## Abstract

**Background:**

The aim of this present study was to compare the prevalence and type of trauma experienced by community sample with the outpatient sample with mental disorders.

**Methods:**

A total of 354 outpatients, aged 14–35 years old, with mood disorders, schizophrenia and other psychotic disorders, adjustment disorder and anxiety disorder were recruited from a tertiary psychiatric hospital. A total of 100 healthy controls were recruited from the Singapore general population by snowballing. The Childhood Trauma Questionnaire-Short Form (CTQ-SF) designed to measure childhood trauma and the severity (e.g., physical abuse, emotional abuse, sexual abuse, physical neglect and emotional neglect) was administered to participants. Socio-demographic and clinical characteristics were obtained from interviews with the participants and from outpatients’ medical records, respectively. Independent sample *t* tests and Chi-square tests were used to investigate the differences between the outpatient and community samples.

**Results:**

Overall the CTQ-SF total and domain scores indicated that outpatient sample experienced higher rate of traumatic life events in childhood than community sample. Two most reported trauma types were emotional abuse (*n* = 81, 59.1%) and physical neglect (*n* = 74, 54%) reported by the mood disorder group. In the community sample, emotional neglect (*n* = 46, 46%) and physical neglect (*n* = 18, 18%) were the most commonly reported trauma type. Overall outpatient sample (*n* = 80, 22.6%) and community sample (*n* = 28, 28%) reported at least one type of trauma.

**Conclusion:**

The findings indicate higher rates of CTQ-SF total and domain scores in outpatient sample demonstrating a higher rate of traumatic life events in childhood compared to community sample. Further research in childhood trauma is needed to improve the knowledge in psychiatric clinic practices.

## Background

The literature has identified the association between adverse childhood experiences (ACEs) such as traumatic events or maltreatment and their harmful impact on adolescent and adult psychopathology across life [1, 2]. A survey among 21 countries including Belgium, Japan, USA, South Africa and China by the World Mental Health (WMH) Initiative found that among the total of 51,945 adults (age 18 and older) recruited, almost 40 percent of the population had adverse childhood experiences (ACEs) [3]. ACEs comprise exposure to chronic environmental stressors such as domestic violence, childhood maltreatment (e.g., emotional, physical or sexual abuse, etc.) and interpersonal loss (e.g., parental mental illness, parental divorce, or parental death) as a child (17 years and below) [4–7]. Children exposed to severe maltreatment and trauma during their early childhood are at a higher risk of early onset of mental disorders [8], increased health-harming behaviors [9, 10], poorer social adjustment, functioning, educational and employment outcomes as adolescents and adults [6, 11–15]. Further analyses by Kessler, McLaughlin [3] suggested that 29.8% of incidences of mental disorder in patients may be associated with adversities experienced in their childhood. For instance, 75.6% of chronically depressed patients aged between 20 and 60 years old in Germany reported trauma experienced in childhood and 37% reported multiple types of childhood trauma [16]. In general, studies have reported a significantly higher prevalence of childhood trauma in patients with mental disorders, emphasizing the risk of traumatic experiences in subsequent psychopathology [13, 17–19].

Childhood trauma has been well documented as a potential risk factor for psychosis [20, 21]. A literature review by Read, van Os [22] found high rates of childhood sexual and physical abuse among patients with psychosis. More specifically, the risk of developing psychotic disorder was 15 times higher for children who were sexually abused as compared to the general population [23]. Conversely, those exposed to childhood sexual and emotional abuse have reported higher psychotic symptoms, suicidal behavior, delusions and hallucinations [24]. As psychotic disorders are highly heritable [25], childhood trauma potentially has a role in interacting with genetic factors in the development of psychiatric disorders [26].

Childhood trauma has been found to contribute to the early onset and severity of bipolar disorder, resulting in poorer clinical outcomes, higher prevalence of a faster cycling pattern and suicide attempts [27, 28]. The prevalence of childhood abuse was 49% in bipolar patients [29]. In particular, Hyun, Friedman [30] reported a strong association between mood disorders and childhood sexual abuse.

Although little research has examined the link between childhood trauma and anxiety disorders, a few studies [31–33] have proposed theories suggesting specific pathways. Cognitive theories suggest that life experiences shape maladaptive schemas which in turn influence adults’ attachment style and interpersonal relationships [34, 35]. Repeated early negative experiences (e.g., emotional abuse, criticism, dysfunctional parental relationship, etc.) directly lead to the development of cognitive vulnerability in an individual [34]. Studies have reported that victims of childhood trauma experience intimacy dysfunction, social adjustment difficulties and lower relationship quality [36–38]. Additionally, Kendler et al. [39] documented how patients with depression and anxiety disorders who experienced childhood sexual abuse have significantly higher load of all types of childhood adversities leading to worse pre-treatment social functioning, earlier age of onset, higher suicidal ideation, chronicity of depression and recurrent episodes [38, 40–43].

Singapore is a small independent island situated in South East Asia with a multi-ethnic population of 5.6 million (74.3% Chinese, 13.4% Malay, 9.0% Indian and 3.2% others) [44]. Studies about physical and sexual abuse victims in Singapore are mainly on children and youth [45, 46]. Majority of the sexual abuse perpetrators in Singapore were non-caregivers, while physical abuse perpetrators were the parents of the child victims [46, 47]. A comparison study reported higher psychological symptoms in Singapore female college students who had a history of child sexual abuse as compared to the non-abused sample and US female sample [48]. However, research in Singapore on the prevalence and impact of childhood trauma in patients with mental disorders is still limited. Therefore, the overall aim of the present study is to investigate the prevalence of childhood trauma among the outpatients with mental disorders receiving treatment in a tertiary psychiatric institute and compare it with the prevalence and type of trauma experienced by a community sample without self-reported mental illness.

## Methods

### Participant

A total of 354 outpatients seeking treatment at the outpatient clinics of the Institute of Mental Health (IMH), Singapore, with a clinical diagnosis of mood disorder, schizophrenia and other psychotic disorder, adjustment disorder and anxiety disorder were recruited. Inclusion criteria for outpatients included: (1) Singaporean or Permanent Resident aged 14–35 years belonging to Chinese, Malay, Indian and other ethnic groups; (2) literate in English language. Exclusion criteria included: (1) those with intellectual disabilities; (2) unable to read English; (3) patients attending the clinic for their first visit. Additionally, a total of 100 healthy controls were recruited from the Singapore general population by a mix of convenience and snowballing sampling. Inclusion criteria for healthy controls included: (1) Singaporean or Permanent Resident aged 14–35 years belonging to Chinese, Malay, Indian and other ethnic groups; (2) literate in English language. The age range considered for our sample was based on two local definitions of youth: the Children and Young Persons Act [49], which defines a young person as 14–16 years old, and the National Youth Council Singapore [50] which defines youths as those aged 15–35 years.

### Procedure

From October 2015 to June 2016, outpatients with mental disorders were recruited at the child and adult outpatient clinics in IMH. The study was approved by the Domain Specific Review Board of the National Healthcare Group, Singapore, and relevant clinicians were informed of the study. Participants either volunteered by responding to posters in the outpatient clinics or they were referred by clinicians who were study team members. Although a waiver of parental consent was granted for participants less than 21 years of age, researchers explained the study to the parents who accompanied them. Nonetheless, written informed consent was obtained from all participants. The research team also confirmed the outpatient’s history by accessing the medical records after obtaining written informed consent. The researchers ensured that the self-administered questionnaires were completed independently by the participants.

## Instruments

### Socio-demographic questionnaire

Socio-demographic information (e.g., age, gender, ethnicity, marital status, diagnosis and educational level) were collected using a structured questionnaire.

### Childhood Trauma Questionnaire-Short Form (CTQ-SF)

The CTQ-SF is a 28-item validated instrument, designed to measure childhood adversity and severity of childhood abuse and neglect suffered [51]. The CTQ-SF includes five subscales: physical abuse (PA) (e.g., “I was punished with a belt, a board, a cord, or some other hard object”), emotional abuse (EA) (e.g., “People in my family said hurtful or insulting things to me”), sexual abuse (SA) (e.g., “Someone tried to make me do sexual things or watch sexual things”), physical neglect (PN) (e.g., “I didn’t have enough to eat”) and emotional neglect (EN) (e.g., “I felt loved”) [51]. Each subscale was measured by rating 5 items on a 5-point Likert scale, ranging from 1 (never true) to 5 (very often true). The scoring responses for CTQ-SF subscales were analyzed together with an overall test of childhood trauma, with all the subscales summed together to classify childhood trauma history among participants in their respective categories. Cutoff scores for CTQ-SF subscales were EA score ≥ 13, PA score ≥ 10, SA score ≥ 8, EN score ≥ 15 and PN score ≥ 10 [17, 52]. The CTQ-SF has been demonstrated to be a reliable and valid tool with acceptable psychometric properties overall for assessing childhood adversity (Cronbach *α* > 0.80) [53–55]. Although local adaptation and validation study on the CTQ-SF were not found, CTQ-SF has been used in local clinical settings and other studies concluded that the scale had an acceptable psychometric property [17].

## Statistical analyses

### Statistical analysis

Statistical analyses were performed using SPSS, version 23. Descriptive statistics and frequency distribution were tabulated for the profile of both samples and the responses of the questions. After controlling for confounding variables, independent sample *t* tests and Chi-square tests were used to compare the differences in means and proportions of continuous and categorical variables between the outpatient and community samples, respectively. Statistical significance was reported at *P* < 0.05 (2-tailed) throughout the study.

## Results

### Sample characteristics

Socio-demographics characteristics of the participants are shown in Table 1. A total of 354 participants consisting 169 males and 185 females, diagnosed with mood disorders (30.2%), psychotic disorders (20%), adjustment disorder (14.3%) and anxiety disorders (13.4%) were recruited for the outpatient sample. Majority of the participants were of Chinese ethnicity (67.8%) and had secondary/junior college/pre-university education (41.6%). A total of 100 participants were recruited for the community sample of which 45 were males and 55 females.

**Table 1:** Socio-demographic characteristics

Variables	Overall sample (*n* = 454)	Outpatient sample (*n* = 354)	Community sample (*n* = 100)
Age, mean ± SD	23.6 ± 6.0	23.7 ± 6.04	23.4 ± 5.89
Sex, *N* (%)			
Male	214 (47.1)	169 (47.7)	45 (45.0)
Female	240 (52.9)	185 (52.3)	55 (55.0)
Ethnicity, *N* (%)			
Chinese	308 (67.8)	285 (71.2)	60 (60.0)
Malay	82 (18.1)	71 (17.7)	15 (15.0)
Indian	51 (11.2)	30 (7.50)	22 (22.0)
Others	13 (2.9)	14 (3.50)	3 (3.00)
Marital status, *N* (%)			
Never married	60 (13.2)	41 (11.6)	19 (19.0)
Ever married	394 (86.8)	313 (88.4)	81 (81.0)
Religion, *N* (%)			
Buddhism	103 (22.7)	76 (21.5)	27 (27.0)
Hinduism	33 (7.30)	15 (4.20)	18 (18.0)
Islam	91 (20.0)	71 (20.1)	20 (20.0)
Taoism	19 (4.20)	12 (3.4)	7 (7.0)
Others	103 (22.7)	90 (25.4)	13 (13.0)
Christianity	105 (23.1)	90 (25.4)	15 (15.0)
Diagnosis, *N* (%)			
Mood disorders		137 (30.2)	
Schizophrenia or Psychotic disorders		91 (20.0)	
Adjustment disorders		65 (14.3)	
Anxiety disorders		61 (13.4)	
Years of education, *N* (%)			
Some formal/primary education	34 (7.50)	23 (6.50)	11 (11.0)
Secondary/‘O’/‘N’ level	156 (34.4)	132 (37.3)	24 (24.0)
‘A’ level/diploma/pre-university	189 (41.6)	164 (46.3)	25 (25.0)
Degree/postgrad degree	75 (16.5)	35 (9.90)	40 (40.0)

### Prevalence of childhood trauma

**Fig. 1:**
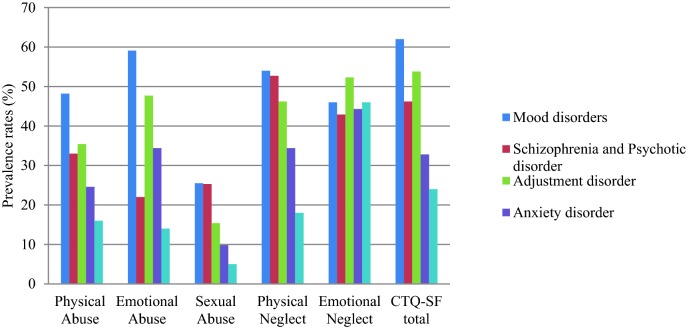
Prevalence of childhood trauma in psychiatric outpatients

The mean CTQ scores for the outpatient sample were 51.9 (SD = 14.8) and 44.3 (SD = 9.6) for community sample (Table 2). The prevalence of childhood trauma in each diagnostic group is presented in Table 3 and in Fig. 1. In the mood disorder group, the two most reported trauma types were emotional abuse (*n* = 81, 59.1%) and physical neglect (*n* = 74, 54%). Besides that, emotional neglect was also widely reported in psychotic disorder (*n* = 39, 43%) and adjustment disorder (*n* = 34, 52.3%) groups. Similarly, emotional neglect (*n* = 46, 46%) and physical neglect (*n* = 18, 18%) were the most commonly reported trauma type in the community sample.

**Table 2:** Mean CTQ scores between outpatient and community sample

	Outpatient sample (*n* = 354)	Community sample (*n* = 100)	*P* value*
CTQ-SF scores, mean ± SD			
Physical abuse (PA)	9.4 ± 5.3	7.3 ± 3.1	< 0.01
Emotional abuse (EA)	12.2 ± 5.6	9.1 ± 3.7	< 0.01
Sexual abuse (SA)	6.9 ± 4.4	5.4 ± 2.1	< 0.01
Physical neglect (PN)	9.5 ± 2.8	8.1 ± 1.9	0.62
Emotional neglect (EN)	14.2 ± 4.8	14.4 ± 3.6	< 0.01
CTQ-SF total	51.9 ± 14.8	44.3 ± 9.6	< 0.01

*P values were derived from independent *t* test and Chi-square test between the outpatient and community samples, respectively

**Table 3:** Prevalence of childhood trauma in psychiatric outpatients

Variables	Mood disorders (*n* = 137)		Schizophrenia or Psychotic disorder (n = 91)		Adjustment disorder (*n* = 65)		Anxiety disorder (*n* = 61)		Overall outpatient sample (*n* = 354)		Community sample (*n* = 100)	
	*N*	%	*N*	%	*N*	%	*N*	%	*N*	%	*N*	%
CTQ-SF scores												
Physical abuse (PA)	66	48.2	30	33.0	23	35.4	15	24.6	134	37.9	16	16.0
Emotional abuse (EA)	81	59.1	20	22.0	31	47.7	21	34.4	153	43.2	14	14.0
Sexual abuse (SA)	35	25.5	23	25.3	10	15.4	6	9.80	74	20.9	5	5.00
Physical neglect (PN)	74	54.0	48	52.7	30	46.2	21	34.4	173	48.9	18	18.0
Emotional neglect (EN)	63	46.0	39	42.9	34	52.3	27	44.3	163	46	46	46.0
CTQ-SF total	85	62.0	42	46.2	35	53.8	20	32.8	182	51.4	24	24.0

### Number of trauma types among outpatient and community sample

The number of existing trauma types was also calculated as shown in Table 4. Twenty (14.6%) outpatients with mood disorder, 20 (21.9%) with schizophrenia or psychotic disorder, 22 (33.9%) with adjustment disorder, 18 (29.5%) with anxiety disorder and 28 (28%) participants in the community sample reported at least one type of trauma. Among outpatients, 34 (24.8%) with mood disorder, 15 (16.5%) with schizophrenia or psychotic disorder, 14 (21.5%) with adjustment disorder, 13 (21.3%) with anxiety disorder and 18 (18%) from the community sample reported more than one type of trauma (Table 4).

**Table 4:** Number of trauma types among outpatient and community sample

Variables	Mood disorders		Schizophrenia or psychotic disorder		Adjustment disorder		Anxiety disorder		Overall outpatient sample		Community sample	
	*N*	%	*N*	%	*N*	%	*N*	%	*N*	%	*N*	%
No trauma	22	16.1	26	28.6	7	10.8	17	27.9	72	20.3	43	43
1 type of trauma	20	14.6	20	21.9	22	33.9	18	29.5	80	22.6	28	28
2 types of trauma	34	24.8	15	16.5	14	21.5	13	21.3	76	21.5	18	18
3 types of trauma	26	18.9	14	15.4	12	18.5	8	13.1	60	16.9	9	9
4 types of trauma	22	16.1	12	13.2	8	12.3	3	4.9	45	12.7	2	2
5 types of trauma	13	9.5	4	4.4	2	3.1	2	3.3	21	5.9	0	0

## Discussion

This study investigated the prevalence of childhood trauma among outpatients with a clinical diagnosis of mood, schizophrenia, psychotic, adjustment and anxiety disorders in Singapore. The study showed higher rates of CTQ-SF total and domain scores in outpatient sample indicating the higher rate of traumatic life events in childhood. This finding is consistent with other studies that showed that childhood trauma is more prevalent among individuals with mental illness than healthy individuals [56–58].

The prevalence rates of childhood trauma in the present study are somewhat higher than previous studies’ prevalence estimate in depression (26.2%) and schizophrenia (22.2%) groups [17, 59]. One explanation for the differences between our results and those of previous studies could be the cultural and methodological differences (e.g., varying cutoff points of CTQ-SF subscales) that affect the occurrence of childhood trauma. Additionally, considering the small sample size in the current study, sampling error might have also contributed to the variation in prevalence rates among the studies.

While PN and EN were the two most reported types of trauma among those with psychotic, adjustment and anxiety disorders, EA and PN were the most frequently reported types of trauma in the mood disorder sample. Exposure to neglect and emotional abuse during childhood influences the mental development of adolescents [60] and is associated in particular with mood and personality disorders during adulthood [61]. Additionally, neglect specifically has been shown to increase the risk of experiencing atypical neurodevelopment [19, 62] and positive psychotic symptoms [19].

In accordance with other studies, this study provides further evidence of the higher prevalence of SA in outpatients with mental illness. Specifically, there was a higher incidence of SA reported among mood and schizophrenia or psychotic disorders when compared with other diagnostic groups in the outpatient sample. Childhood sexual abuse (CSA) interrupts the development of a child’s sense of identity causing difficulties in interpersonal skills and emotional regulation which influence the development of different types of mood [52] and psychiatric disorders [13, 63–65]. Hence, sexually abused victims are more likely to report a lifetime history of depression and have a higher risk of developing psychotic disorder [63, 66, 67].

It is noteworthy that EN was the only type of trauma that was reported equally in both outpatient and community samples—a prevalence of 46% which was similar to the mood disorder sample. Some cultural and social factors need to be considered. One explanation could be the cultural differences between Western and Asian countries particularly in parenting styles [68]. Studies have variously described parenting styles in Asian culture as “authoritarian,” “strict” and “lacking in expressivity” as compared to authoritative parenting styles (e.g., high in support and moderate control) commonly seen in Western culture [68–70]. In Asian cultures, parents often express their love for children by providing resources for their children’s physical needs or through sacrifices [69] and physical or corporal punishment which is often the common parental disciplinary technique in some traditional families [71, 72]. Parental expressivity such as acceptance and care in Asian culture are shown through “instrumental support” (e.g., clothing, food, schooling, etc.) rather than verbal expressions (e.g., “I love you,” hugs and praising) [69]. In addition, with the increased influences of Westernization in the Asian cultures [73], the younger generation may perceive their parents’ parenting practices as devoid of emotional warmth [74], which could lead to the perception of emotional neglect, hence the higher reported prevalence rate in the present study. Nevertheless, authoritative parenting is not necessarily more damaging or disadvantageous than authoritarian parenting given the different values across cultures.

The present study also investigated the number of trauma types that were reported in each sample. The frequency of cases with multiple traumas was higher among the four outpatient samples than the community sample, a finding consistent with some previous studies [16, 17]. In addition, outpatients with mood disorder reported a higher prevalence of multiple traumas when compared with other diagnostic groups which was also reported by Etain, Aas [32] in a study among outpatients with bipolar disorder. Mainly, multiple childhood trauma experiences are assumed to have a substantial influence on the risk of development and severity of mental illness [75]. For example, in the longitudinal study by Widom, DuMont [76], children who experienced multiple types of abuse or neglect had a higher risk of developing depression in later life.

## Limitations

The present study is a preliminary investigation of the prevalence and severity of childhood trauma among outpatients with mental disorders in Singapore, and thus, there are some limitations. Firstly, the study may not be sufficiently representative because outpatients with disorders that are usually diagnosed in childhood (e.g., autism spectrum disorder, attention deficit hyperactivity disorder (ADHD), attention deficit disorder (ADD) with hyperactivity, etc.) were excluded. Secondly, socio-demographic characteristics including age, marital status, religion and years of education were significantly different between both outpatient and community sample, which may result in differences. Thirdly, local adaptation and validation study on the CTQ-SF were not found for the population studied. Lastly, only participants who were literate in English and capable of self-administering the CTQ-SF scale were recruited.

## Conclusion

In summary, this study investigated the prevalence of childhood trauma in an outpatient sample with mental disorders among a clinical and nonclinical population in Singapore. Consistent with previous studies, many individuals with mental disorders had experienced childhood trauma and also multiple trauma types compared with healthy individuals. The present study suggests that childhood trauma needs more attention, particularly in psychiatric clinical practices and scientific research. Such information is important as it contributes to the public education on the negative impact of childhood adversity toward a child’s mental health development and the effective approaches to treatment.

## Data Availability

Data supporting the findings are available upon request. Please contact the Principal Investigator, Ms. Shazana Shahwan (Shazana_MOHAMED_SHAHWAN@imh.com.sg), for data availability.

## Abbreviation

CTQ-SF: Childhood Trauma Questionnaire-Short Form (CTQ-SF)

## References

[1] Hughes K, Bellis MA, Hardcastle KA, Sethi D, Butchart A, Mikton C (2017). The effect of multiple adverse childhood experiences on health: a systematic review and meta-analysis. Lancet Public Health.

[2] Busso DS, McLaughlin KA, Brueck S, Peverill M, Gold AL, Sheridan MA (2017). Child abuse, neural structure, and adolescent psychopathology: a longitudinal study. J Am Acad Child Adolesc Psychiatry.

[3] Kessler RC, McLaughlin KA, Green JG, Gruber MJ, Sampson NA, Zaslavsky AM (2010). Childhood adversities and adult psychopathology in the WHO World Mental. Br J Psychiatry.

[4] Bellis MA, Hughes K, Hardcastle K, Ashton K, Ford K, Quigg Z (2017). The impact of adverse childhood experiences on health service use across the life course using a retrospective cohort study. J Health Serv Res Policy.

[5] De Bellis MD, Zisk A (2014). The biological effects of childhood trauma. Child Adolesc Psychiatr Clin N Am.

[6] Pechtel P, Pizzagalli DA (2011). Effects of early life stress on cognitive and affective function: an integrated review of human literature. Psychopharmacology.

[7] Behere AP, Basnet P, Campbell P (2017). Effects of family structure on mental health of children: a preliminary study. Indian J Psychol Med.

[8] Teicher MH, Samson JA, Anderson CM, Ohashi K (2016). The effects of childhood maltreatment on brain structure, function and connectivity. Nat Rev Neurosci.

[9] Bellis MA, Hughes K, Leckenby N, Perkins C, Lowey H (2014). National household survey of adverse childhood experiences and their relationship with resilience to health-harming behaviors in England. BMC Med.

[10] Bellis MA, Hughes K, Leckenby N, Jones L, Baban A, Kachaeva M (2014). Adverse childhood experiences and associations with health-harming behaviours in young adults: surveys in eight eastern European countries. Bull World Health Organ.

[11] DeRosse P, Nitzburg GC, Kompancaril B, Malhotra AK (2014). The relation between childhood maltreatment and psychosis in patients with schizophrenia and non-psychiatric controls. Schizophr Res.

[12] Bellis MA, Hughes K, Leckenby N, Hardcastle KA, Perkins C, Lowey H (2015). Measuring mortality and the burden of adult disease associated with adverse childhood experiences in England: a national survey. J Public Health.

[13] Larsson S, Andreassen OA, Aas M, Rossberg JI, Mork E, Steen NE (2013). High prevalence of childhood trauma in patients with schizophrenia spectrum and affective disorder. Compr Psychiatry.

[14] Liu Y, Croft JB, Chapman DP, Perry GS, Greenlund KJ, Zhao G (2013). Relationship between adverse childhood experiences and unemployment among adults from five US states. Soc Psychiatry Psychiatr Epidemiol.

[15] Weber K, Rockstroh B, Borgelt J, Awiszus B, Popov T, Hoffmann K (2008). Stress load during childhood affects psychopathology in psychiatric patients. BMC Psychiatry.

[16] Negele A, Kaufhold J, Kallenbach L, Leuzinger-Bohleber M (2015). Childhood trauma and its relation to chronic depression in adulthood. Depress Res Treat.

[17] Xie P, Wu K, Zheng Y, Guo Y, Yang Y, He J (2018). Prevalence of childhood trauma and correlations between childhood trauma, suicidal ideation, and social support in patients with depression, bipolar disorder, and schizophrenia in southern China. J Affect Disord.

[18] Reeder FD, Husain N, Rhouma A, Haddad PM, Munshi T, Naeem F (2017). The relationship between childhood trauma and adult psychosis in a UK early intervention service: results of a retrospective case note study. Neuropsychiatr Dis Treat.

[19] Duhig M, Patterson S, Connell M, Foley S, Capra C, Dark F (2015). The prevalence and correlates of childhood trauma in patients with early psychosis. Aust N Z J Psychiatry.

[20] Aas M, Andreassen OA, Aminoff SR, Færden A, Romm KL, Nesvåg R (2016). A history of childhood trauma is associated with slower improvement rates: findings from a one-year follow-up study of patients with a first-episode psychosis. BMC Psychiatry.

[21] Thompson AD, Nelson B, Yuen HP, Lin A, Amminger GP, McGorry PD (2014). Sexual trauma increases the risk of developing psychosis in an ultra high-risk “prodromal” population. Schizophr Bull.

[22] Read J, van Os J, Morrison AP, Ross CA (2005). Childhood trauma, psychosis and schizophrenia: a literature review with theoretical and clinical implications. Acta Psychiatr Scand.

[23] Cutajar MC, Mullen PE, Ogloff JP, Thomas SD, Wells DL, Spataro J (2010). Schizophrenia and other psychotic disorders in a cohort of sexually abused children. Arch Gen Psychiatry.

[24] Ucok A, Bikmaz S (2007). The effects of childhood trauma in patients with first-episode schizophrenia. Acta Psychiatr Scand.

[25] Cardno AG, Marshall EJ, Coid B, Macdonald AM, Ribchester TR, Davies NJ (1999). Heritability estimates for psychotic disorders: the Maudsley twin psychosis series. Arch Gen Psychiatry.

[26] Kim JS, Lee SH (2016). Influence of interactions between genes and childhood trauma on refractoriness in psychiatric disorders. Prog Neuropsychopharmacol Biol Psychiatry.

[27] Garno JL, Gunawardane N, Goldberg JF (2008). Predictors of trait aggression in bipolar disorder. Bipolar Disord.

[28] Leverich GS, Altshuler LL, Frye MA, Suppes T, Keck PE, McElroy SL (2003). Factors associated with suicide attempts in 648 patients with bipolar disorder in the Stanley Foundation Bipolar Network. J Clin Psychiatry.

[29] Garno JL, Goldberg JF, Ramirez PM, Ritzler BA (2005). Impact of childhood abuse on the clinical course of bipolar disorder. Br J Psychiatry.

[30] Hyun M, Friedman SD, Dunner DL (2000). Relationship of childhood physical and sexual abuse to adult bipolar disorder. Bipolar Disord.

[31] Luby JL, Gaffrey MS, Tillman R, April LM, Belden AC (2014). Trajectories of preschool disorders to full DSM depression at school age and early adolescence: continuity of preschool depression. Am J Psychiatry.

[32] Etain B, Aas M, Andreassen OA, Lorentzen S, Dieset I, Gard S (2013). Childhood trauma is associated with severe clinical characteristics of bipolar disorders. J Clin Psychiatry.

[33] Huh HJ, Kim S-Y, Yu JJ, Chae J-H (2014). Childhood trauma and adult interpersonal relationship problems in patients with depression and anxiety disorders. Ann Gen Psychiatry.

[34] Liu RT (2017). Childhood adversities and depression in adulthood: current findings and future directions. Clin Psychol Sci Pract.

[35] Mason O, Platts H, Tyson M (2005). Early maladaptive schemas and adult attachment in a UK clinical population. Psychol Psychother Theory Res Pract.

[36] Davis JL, Petretic-Jackson PA, Ting L (2001). Intimacy dysfunction and trauma symptomatology: long-term correlates of different types of child abuse. J Trauma Stress.

[37] Ducharme J, Koverola C, Battle P (1997). Intimacy development: the influence of abuse and gender. J Interpers Violence.

[38] Huh HJ, Kim S-Y, Yu JJ, Chae J-H (2014). Childhood trauma and adult interpersonal relationship problems in patients with depression and anxiety disorders. Ann Gen Psychiatry.

[39] Kendler KS, Bulik CM, Silberg J, Hettema JM, Myers J, Prescott CA (2000). Childhood sexual abuse and adult psychiatric and substance use disorders in women: an epidemiological and cotwin control analysis. Arch Gen Psychiatry.

[40] Weinberger MI, Sirey JA, Bruce ML, Heo M, Papademetriou E, Meyers BS (2008). Predictors of major depression six months after admission for outpatient treatment. Psychiatr Serv.

[41] Teicher MH, Samson JA, Polcari A, Andersen SL (2009). Length of time between onset of childhood sexual abuse and emergence of depression in a young adult sample. J Clin Psychiatry.

[42] Wiersma JE, Hovens JG, van Oppen P, Giltay EJ, van Schaik DJ, Beekman AT (2009). The importance of childhood trauma and childhood life events for chronicity of depression in adults. J Clin Psychiatry.

[43] Withers AC, Tarasoff JM, Stewart JW (2013). Is depression with atypical features associated with trauma history?. J Clin Psychiatry.

[44] Singapore DoS. Populations trends 2018. Department of Statistics MoTI, Republic of Singapore; 2018. Report No. ISSN 2591-8028.

[45] Tong CK, Elliott MJ, Tan MEHP (2019). Public perceptions of child abuse and neglect in Singapore.

[46] Chu CM, Thomas SDM, Ng VPY (2009). Childhood abuse and delinquency: a descriptive study of institutionalized female youth in Singapore. Psychiatry Psychol Law.

[47] Tong CK, Elliott JM, Tan P (1996). Public perceptions of child abuse and neglect in Singapore.

[48] Back SE, Jackson JL, Fitzgerald M, Shaffer A, Salstrom S, Osman MM (2003). Child sexual and physical abuse among college students in Singapore and the United States. Child Abuse Negl.

[49] Children And Young Persons Act (Chapter 38). Singapore statutes online PLUS; 1993.

[50] Ng IYH. Youth Scope: youth participation in Singapore, Chap 2. In: Journal of youth research studies in Singapore; 2012, p. 18–22. Available from: http://www.youthpolicy.org/library/wp-content/uploads/library/Singapore_2012_Youth_Scope_Report_eng.pdf

[51] Bernstein DP, Stein JA, Newcomb MD, Walker E, Pogge D, Ahluvalia T (2003). Development and validation of a brief screening version of the childhood trauma questionnaire. Child Abuse Negl.

[52] Jansen K, Cardoso TA, Fries GR, Branco JC, Silva RA, Kauer-Sant’Anna M (2016). Childhood trauma, family history, and their association with mood disorders in early adulthood. Acta Psychiatr Scand.

[53] Karos K, Niederstrasser N, Abidi L, Bernstein DP, Bader K (2014). Factor structure, reliability, and known groups validity of the German version of the childhood trauma questionnaire (short-form) in Swiss patients and nonpatients. J Child Sex Abuse.

[54] Kim D, Bae H, Han C, Oh HY, Macdonald K (2013). Psychometric properties of the childhood trauma questionnaire-short form (CTQ-SF) in Korean patients with schizophrenia. Schizophr Res.

[55] Garrusi B, Nakhaee N (2009). Validity and reliability of a Persian version of the childhood trauma questionnaire. Psychol Rep.

[56] Bonoldi I, Simeone E, Rocchetti M, Codjoe L, Rossi G, Gambi F (2013). Prevalence of self-reported childhood abuse in psychosis: a meta-analysis of retrospective studies. Psychiatry Res.

[57] Fisher HL, Jones PB, Fearon P, Craig TK, Dazzan P, Morgan K (2010). The varying impact of type, timing and frequency of exposure to childhood adversity on its association with adult psychotic disorder. Psychol Med.

[58] Church C, Andreassen OA, Lorentzen S, Melle I, Aas M (2017). Childhood trauma and minimization/denial in people with and without a severe mental disorder. Front Psychol.

[59] Alvarez M-J, Roura P, Osés A, Foguet-Boreu Q, Solà J, Arrufat F (2011). Prevalence and clinical impact of childhood trauma in patients with severe mental disorders. J Nerv Ment Disord..

[60] Mills R, Scott J, Alati R, O’Callaghan M, Najman J, Strathearn L (2013). Child maltreatment and adolescent mental health problems in a large birth cohort. Child Abuse Negl.

[61] Carr C, Martins C, Stingel A, Braga Lemgruber V, Juruena M (2013). The role of early life stress in adult psychiatric disorders: a systematic review according to childhood trauma subtypes. J Nerv Ment Disord.

[62] Glaser D (2014). The effects of child maltreatment on the developing brain. Med Leg J.

[63] Molnar BE, Buka SL, Kessler RC (2001). Child sexual abuse and subsequent psychopathology: results from the national comorbidity survey. Am J Public Health.

[64] Rössler W, Ajdacic-Gross V, Haker H, Rodgers S, Müller M, Hengartner M (2015). Subclinical psychosis syndromes in the general population: results from a large-scale epidemiological survey among residents of the canton of Zurich, Switzerland. Epidemiol Psychiatr Sci.

[65] Sahin S, Yuksel C, Guler J, Karadayi G, Akturan E, Gode E (2013). The history of childhood trauma among individuals with ultra high risk for psychosis is as common as among patients with first-episode schizophrenia. Early Interv Psychiatry.

[66] Bebbington PE, Bhugra D, Brugha T, Singleton N, Farrell M, Jenkins R (2004). Psychosis, victimisation and childhood disadvantage: evidence from the second British national survey of psychiatric morbidity. Br J Psychiatry.

[67] Molnar BE, Berkman LF, Buka SL (2001). Psychopathology, childhood sexual abuse and other childhood adversities: relative links to subsequent suicidal behaviour in the US. Psychol Med.

[68] Ren L, Pope Edwards C (2015). Pathways of influence: Chinese parents’ expectations, parenting styles, and child social competence. Early Child Dev Care.

[69] Institute FM. Cultural differences in parenting practices: what Asian American families can teach us. Frances McClelland Institute; 2010. Contract No. 1.

[70] Ang R, Goh D (2006). Authoritarian parenting style in Asian societies: a cluster-analytic investigation. Contemp Fam Ther.

[71] Choi Y, Kim YS, Kim SY, Park IK (2013). Is Asian American parenting controlling and harsh? Empirical testing of relationships between Korean American and Western parenting measures. Asian Am J Psychol.

[72] Xing X, Zhang H, Shao S, Wang M (2017). Child negative emotionality and parental harsh discipline in Chinese preschoolers: the different mediating roles of maternal and paternal anxiety. Front Psychol.

[73] Wang I-C, Guo L (2013). Introduction to Asian culture(s) and globalization. Comp Lit Cult.

[74] Mousavi SE, Low WY, Hashim AH (2016). Perceived parenting styles and cultural influences in adolescent’s anxiety: a cross-cultural comparison. J Child Fam Stud.

[75] Norman RE, Byambaa M, De R, Butchart A, Scott J, Vos T (2012). The long-term health consequences of child physical abuse, emotional abuse, and neglect: a systematic review and meta-analysis. PLoS Med.

[76] Widom CS, DuMont K, Czaja SJ (2007). A prospective investigation of major depressive disorder and comorbidity in abused and neglected children grown up. Arch Gen Psychiatry.

